# COVID-19 vaccine acceptance among pregnant women worldwide: A systematic review and meta-analysis

**DOI:** 10.1371/journal.pone.0272273

**Published:** 2022-09-28

**Authors:** Milad Azami, Marzieh Parizad Nasirkandy, Hadi Esmaeili Gouvarchin Ghaleh, Reza Ranjbar

**Affiliations:** 1 Faculty of Medicine, Ilam University of Medical Sciences, Ilam, Iran; 2 Department of Obstetrics and Gynecology, School of Medicine, Tabriz University of Medical Sciences, Tabriz, Iran; 3 Applied Virology Research Center, Baqiyatallah University of Medical Sciences, Tehran, Iran; 4 Molecular Biology Research Center, Systems Biology and Poisonings Institute, Baqiyatallah University of Medical Sciences, Tehran, Iran; The University of Jordan, JORDAN

## Abstract

**Background:**

The COVID-19 pandemic has led to the death of many people worldwide. The World Health Organization (WHO) has declared vaccine resistance as one of the greatest health threats in the world even before the COVID-19 epidemic. The aim of this study was to evaluate the acceptance of COVID-19 vaccine in pregnant women.

**Method:**

We performed this systematic review and meta-analysis in accordance with the PRISMA guidelines. We applied the standard search strategy to the PubMed/Medline, Web of Science (ISI), Scopus, Science Direct, Cochrane Library, EMBASE, and EBSCO databases, and the Google Scholar search engine. Heterogeneity between studies was relatively high and therefore meta-analyses were performed based on random effects model with 95% CI using STATA version 16.

**Results:**

In 16 articles with a sample size of 19219 pregnant women, the acceptance of COVID-19 vaccine was estimated 53.46% (95%CI: 47.64%-59.24%). Subgroup analysis was performed based on continent (p = 0.796), data collection method (p = 0.450) and meta-regression based on the month of the study (P<0.001), and only meta-regression was significant based on the month of the study. The effect of some variables such as graviad (OR = 1.02 [95%CI: 0.90–1.16]), maternal age was (OR = 1.02 [95%CI: 0.93–1.11]) and history of influenza vaccination (OR = 0.87 [95%CI: 0.71–1.06]) on COVID-19 vaccine acceptance was evaluated, which was not significant.

**Conclusion:**

The prevalence of COVID-19 vaccine acceptance in pregnant women was 53.46%, which was much lower than the general COVID-19 vaccination. Therefore, necessary interventions should be taken to increase the acceptance of the vaccine, address safety concerns and educate about it.

## 1. Introduction

The coronavirus disease of 2019 (COVID-19) pandemic has led to the death of many people worldwide [1]. High rate of human mortality has created public health challenges, disrupted the supply chain and the economy, and on the other hand has created a comprehensive mental health crisis [2, 3].

Pregnant women and postpartum women are more likely to get infected with COVID-19 and get excessively stressed out about this disease compared to their non-pregnant peers [4]. Research on pregnant women has shown that although the symptoms of the disease and the death rate are similar to those of non-pregnant women, these individuals are at greater risk for severe disease, intensive care admission, and invasive ventilation [5, 6].

COVID-19 in mothers can cause preterm delivery, stillbirth, multiple organ dysfunction syndrome, increased heart rate and fetal distress, premature rupture of membranes, increased cesarean section rate and death [1, 7]. It is also worth noting that COVID-19 pandemic causes fears for the health of the fetus and its health among pregnant women, which significantly affects their well-being [8].

To date, many pregnant women in the United States, Europe, and some Asian countries have been vaccinated against COVID-19 by different vaccines. With the development of vaccines and the global start of vaccination, there is hope for saving more lives and reducing the severe effects of the disease in all population groups. However, due to the emergency use of these vaccines and also lack of clinical information about their effects on pregnancy and fetus, there are ambiguities in this regard [9, 10].

According to a study by Pratama et al. in a review of vaccine safety during pregnancy, Pfizer-BioNTech and Moderna vaccines are effective in preventing infection and are safe for pregnancy and the fetus [11]. These vaccines are made from modified version of a different virus (adenovirus). Previously, similar vaccines using the same viral vector have been tested in all trimesters of pregnancy and have shown no adverse effects on the infant [12]. In another study, evidence of the safety and efficacy of Pfizer BNT162b2 in pregnancy was presented, which showed health benefits for both mother and infant [13].

It is necessary to know the factors affecting vaccine acceptance among different social groups, including pregnant women [14]. Pregnant women often play a key role in getting their children vaccinated. However, the results obtained by studies about COVID-19 vaccine acceptance among pregnant women show contradictory results [1, 15, 16]. The World Health Organization (WHO) has declared vaccine resistance as one of the greatest health threats in the world even before the COVID-19 epidemic [17]. Preliminary research on COVID-19 vaccine acceptance predicts unprecedented challenges for global vaccination [18]. On the other hand, the National Advisory Committee on Immunization (NACI) and the American College of Obstetricians and Gynecologists (ACOG) recommend that pregnant women must be vaccinated. Therefore, the confusion of pregnant women in deciding on the COVID-19 vaccine has hampered its acceptance. So far, various studies have examined vaccine acceptance in the world, which have led to different results [19].

Considering that obtaining an overall estimate can pave the way for health policymakers to accurately estimate the prevalence of COVID-19 vaccine acceptance in pregnant women [20], we conducted a systematic review and meta-analysis to combine the findings from existing studies in this area and provide a clearer picture of its prevalence in the world [21].

## 2. Method

### 2.1. Study protocol

Before beginning this study, in the assessment of protocol registry for systematic reviews, it was found that the review protocol was not recorded in any database. We performed this systematic review and meta-analysis in accordance with the Preferred Reporting Items for Systematic Reviews and Meta-analysis (PRISMA) guidelines [22]. Each of the research stages, including search, selection of articles, data extraction, and qualitative evaluation of selected studies, was performed independently by at least two authors (M.A, R.R, and M.P) and the contradictions were resolved by consensus.

### 2.2. Search strategy

We did our initial literature search in October 2021. We applied the standard search strategy to the PubMed/Medline, Web of Science (ISI), Scopus, Science Direct, Cochrane Library, EMBASE, and EBSCO databases, and the Google Scholar search engine. We reviewed reference lists of identified articles to find other related articles. Searches were last updated in November 2021.

The search was performed using the following Mesh terms: “Coronavirus"[Mesh], "COVID-19"[Mesh], "SARS-CoV-2"[Mesh], "COVID-19 Vaccines"[Mesh], "Pregnancy"[Mesh], and “Pregnant Women” [Mesh]. An example of a combined search in PubMed is as follows: ((“Coronavirus"[Mesh]) OR (“COVID-19"[Mesh] OR "SARS-CoV-2"[Mesh] OR "COVID-19 Vaccines"[Mesh])) AND (("Pregnancy"[Mesh] OR "Pregnant Women"[Mesh])).

### 2.3. Inclusion and exclusion criteria

The included articles had the following inclusion criteria: English articles based on a cross-sectional design with short abstract that examined vaccine acceptance in pregnant women. Articles were excluded if: 1) they used a selective sampling (e.g., interventional trials after group allocation), 2) their samples included groups other than pregnant women, 3) they had sample size of ≤ 50, 4) their subject was not related to our target subject, 5) they were duplicate studies, 6) they were case reports, review articles, congress, letters to the editor without quantitative data, and dissertations, 7) they had low quality in qualitative evaluation, and 8) they did not separate pregnant women from lactating women.

### 2.4. Article selection

Titles and abstracts of all identified reports were reviewed. The full text of the articles was then evaluated based on inclusion and exclusion criteria.

### 2.5. Data extraction

The following data were extracted from each article: First author, year of publication, month of study, study design, number of participants (total, based on pregnancy trimester, based on group, based on history of influenza vaccine injection, based on age [below and above 35 years]), Data collection tools, acceptance of COVID-19 vaccine (total, based on pregnancy trimester, based on history of influenza vaccine injection, based on age) and odds ratio (OR) and 95% confidence interval (CI) for variables.

### 2.6. Quality assessment

The adapted version of Newcastle-Ottawa Scale was used to assess the quality of nonrandomized studies [23]. The maximum attainable score was 9. Three categories were defined for the quality of articles: low quality (score less than 5), medium quality (score 6–7) and high score (score 8–9).

### 2.7. Statistical analysis

Heterogeneity between studies was relatively high and therefore meta-analyses were performed based on random effects model with 95% CI using STATA version 13. Heterogeneity was assessed using I^2^ with thresholds ≥ 25%, ≥ 50% and ≥ 75%, indicating low, medium and high heterogeneity, respectively [24]. We used the OR_s_ index and 95% CI to show the effect of variables such as age, history of influenza vaccine and gravid on COVID-19 vaccine acceptance. Finally, we reported the results as OR and 95% CI. In studies that did not report OR_s_ index and 95% CI, we obtained case and control cases based on the total sample size of each group as well as the rate of COVID-19 vaccine acceptance in each group. Sensitivity analysis examined whether prevalence estimates were influenced by study design. Publication bias was assessed using Begg’s and Egger’s tests [25, 26]. P-value less than 0.05 were considered statistically significant.

## 3. Results

### 3.1. Search results and features of articles

**Fig 1:**
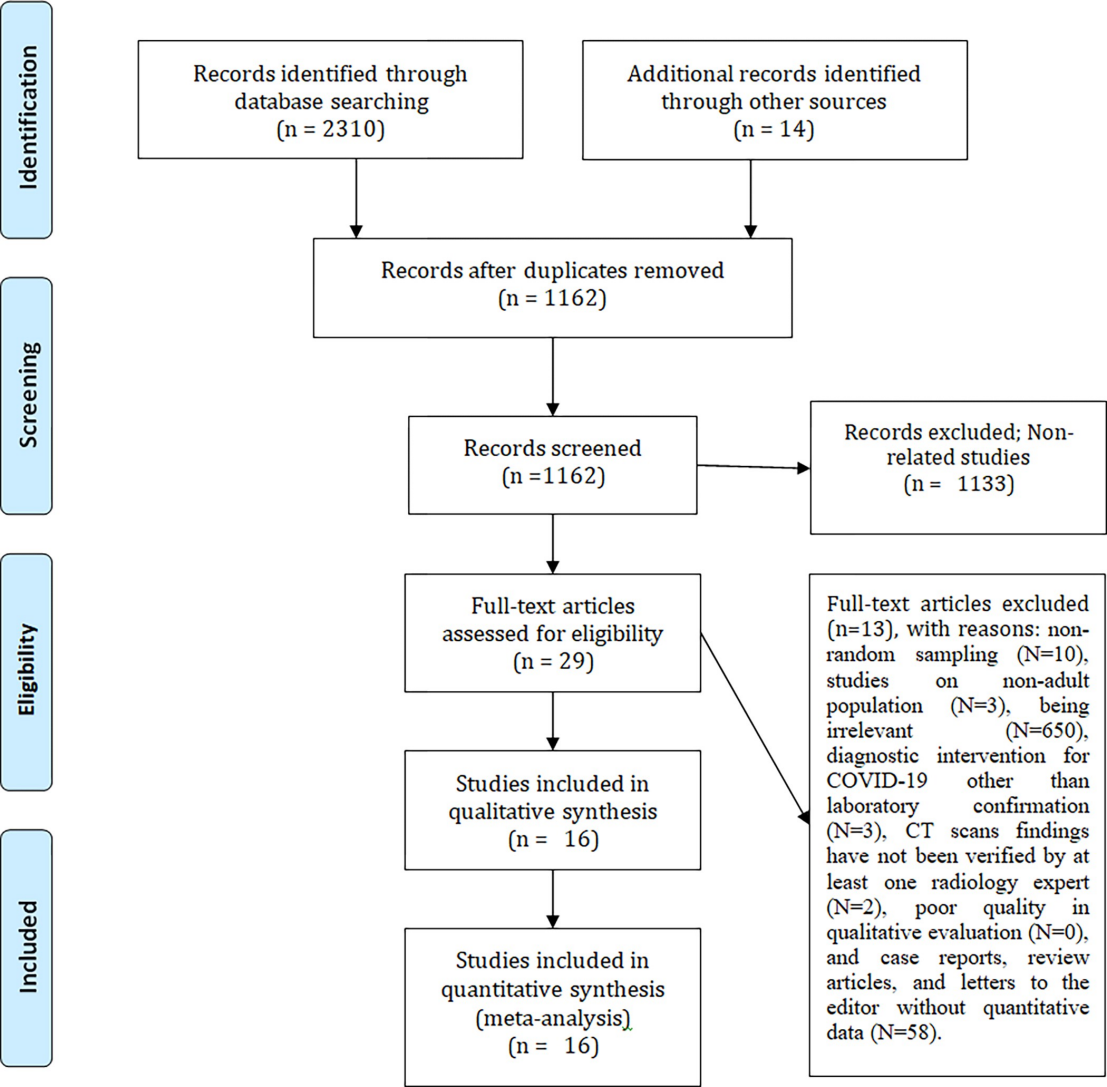
PRISMA flowchart.

In the initial search, 2,324 articles on vaccine acceptance in the world were found. After reviewing the title and abstract, 29 articles were identified as relevant and after reviewing the full text, 13 articles were omitted due to lack of necessary criteria and finally 16 articles were entered into qualitative synthesis and among them, 16 eligible articles (related to acceptance of vaccine in pregnant women) entered the meta-analysis stage (Fig 1). Table 1 shows the specifications of each study.

**Table 1:** Summary of characteristics in studies into a meta-analysis.

Ref.	First author, Published Year	Study duration	Place	Method to collection data	Design			Quality
						All	Accepted	
[27]	Goncu Ayhan S, 2021	January, 2021	Ankara, Turkey	During visit	Cross-sectional	300	111	Medium risk
[28]	Sutton D, 2021	January, 2021	New York	Online survey	Cross-sectional	216	86	Medium risk
[29]	Jayagobi A, 2021	March to May 2021	Singapore	Online survey	Cross-sectional	201	61	Medium risk
[30]	Mose A, 2021	January 2021	Ethiopia	During visit	Cross-sectional	396	280	Medium risk
[31]	Skirrow H, 2021	August to October 2020	UK	Online survey	Cross-sectional	1181	732	Medium risk
[32]	Skjefte M, 2021	October to November 2020	United States (US), India, Brazil, Russia, Spain, Argentina, Colombia, UK, Mexico, Peru, South Africa, Italy, Chile and the Philippines, Australia and New Zealand	Online survey	Cross-sectional	5282	2747	Low risk
[33]	Levy A T, 2021	14th December to 14th January 2020	New York	During visit	Cross-sectional	653	381	Medium risk
[34]	Tao L, 2021	13th November to 27th November 2020	China	During visit	Cross-sectional	1392	1078	Low risk
[35]	Hailemariam SH, 2021	1th February to 1th March 2021	southwest Ethiopia	During visit	Cross-sectional	412	129	Medium risk
[36]	Battarbee A.N, 2021	August to December, 2020	United States	During visit	Cross-sectional	915	374	Low risk
[37]	Hoque A.M, 2020	September to October 2020	Durban	During visit	Cross-sectional	346	217	Medium risk
[15]	Ceulemans M, 2021	June and July 2020	Belgium, Norway, Netherlands, Switzerland, Ireland and UK	Online survey	Cross-sectional	6661	3463	Low risk
[16]	Gencer H, 2021	July and October 2020	Turkey	Online survey	Cross-sectional	152	80	Medium risk
[1, 38]	Mappa I, 2021	January to February 2021	Italy	During visit	Cross-sectional	161	136	Medium risk
[1]	Geoghegan S, 2021	January 2021	Ireland	During visit	Cross-sectional	300	114	Medium risk
[39]	Nguyen LH, 2021	January to February 2021	Vietnam	Online survey	Cross-sectional	651	393	Low risk

NR: not reported, M: Month

* Time for duration of symptoms.

### 3.2. Acceptance of COVID-19 vaccine and sensitivity analysis

**Fig 2:**
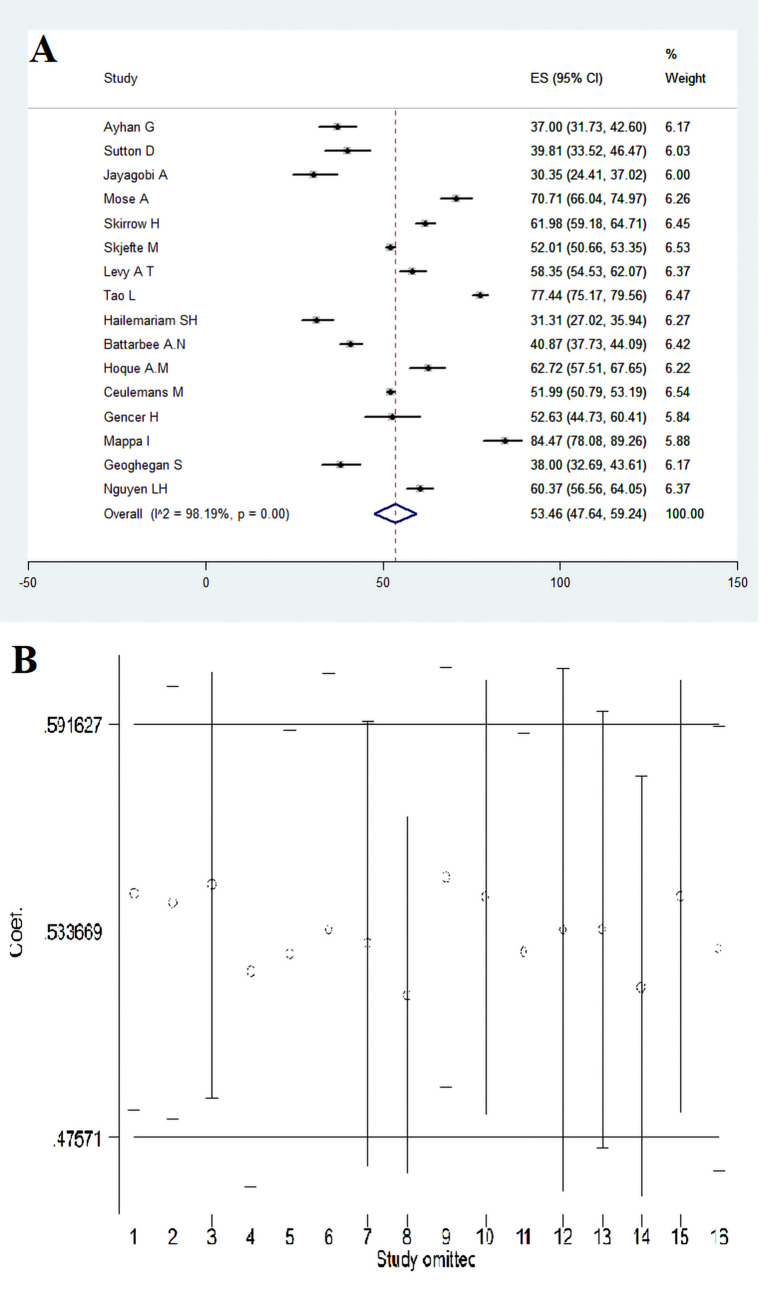
Acceptance of COVID-19 vaccine in pregnant women (A) and sensitivity analysis (B).

In 16 articles with a sample size of 19219 pregnant women, the acceptance of COVID-19 vaccine was estimated at 53.46% (95% CI: 47.64–59.24) (Fig 2A). Furthermore, the sensitivity analysis with the omission of one study at a time showed that the results are still robust and the omission of one study does not affect the overall results (Fig 2B).

### 3.3. Subgroup analysis based on continent

**Fig 3:**
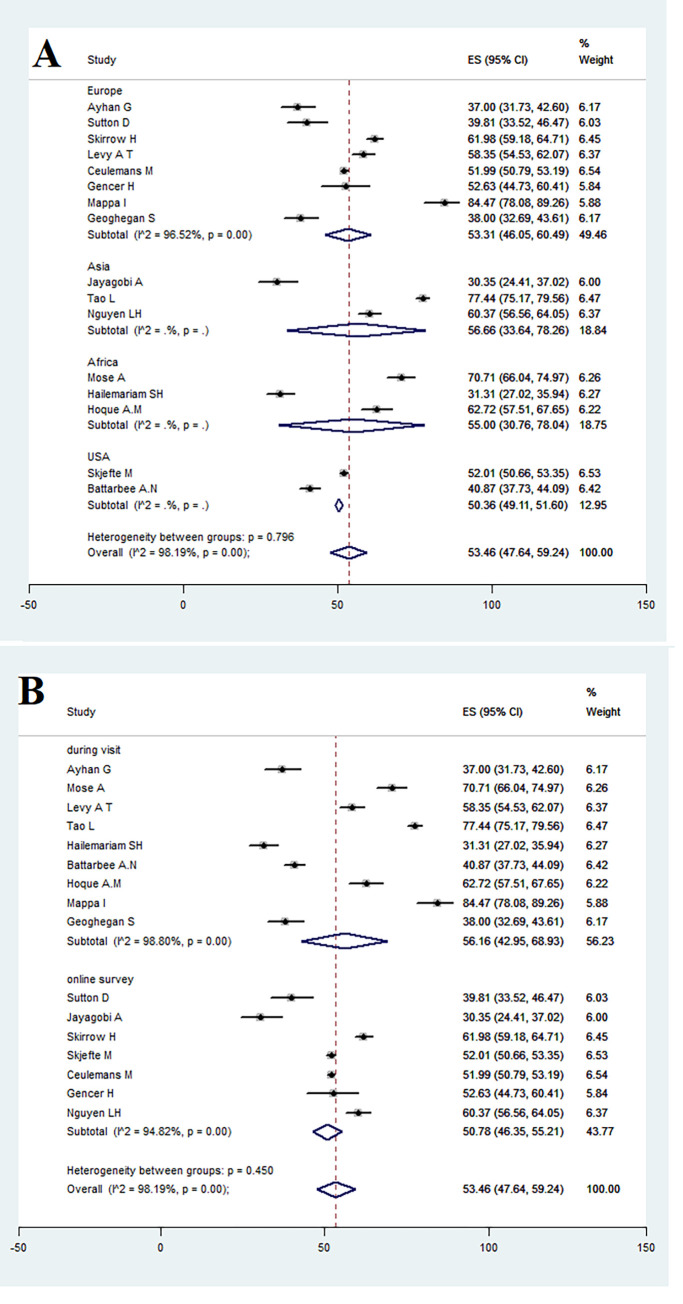
Subgroup analysis of acceptance of COVID-19 vaccine in pregnant women based on continent (A) and (B).

COVID-19 vaccine acceptance among pregnant women in Europe, Asia, Africa, and the United States were respectively estimated as 53.31% (95% CI: 46.05–60.49), 56.66% (95% CI: 33.64–78.26), 55.00% (95% CI: 30.76–78.04), and 50.36% (95% CI: 47.64–59.24) and heterogeneity between subgroups was not significant (p = 0.796) (Fig 3A).

### 3.4. Subgroup analysis based on data collection method

COVID-19 vaccine acceptance among pregnant women was estimated at 56.16% (95% CI: 42.95–68.93) in face-to-face visit and 50.78% (95% CI: 46.35–55.21) in online visit. Heterogeneity was not significant between subgroups (p = 0.450) (Fig 3B).

### 3.5. Effect of maternal age on COVID-19 vaccine acceptance

**Fig 4:**
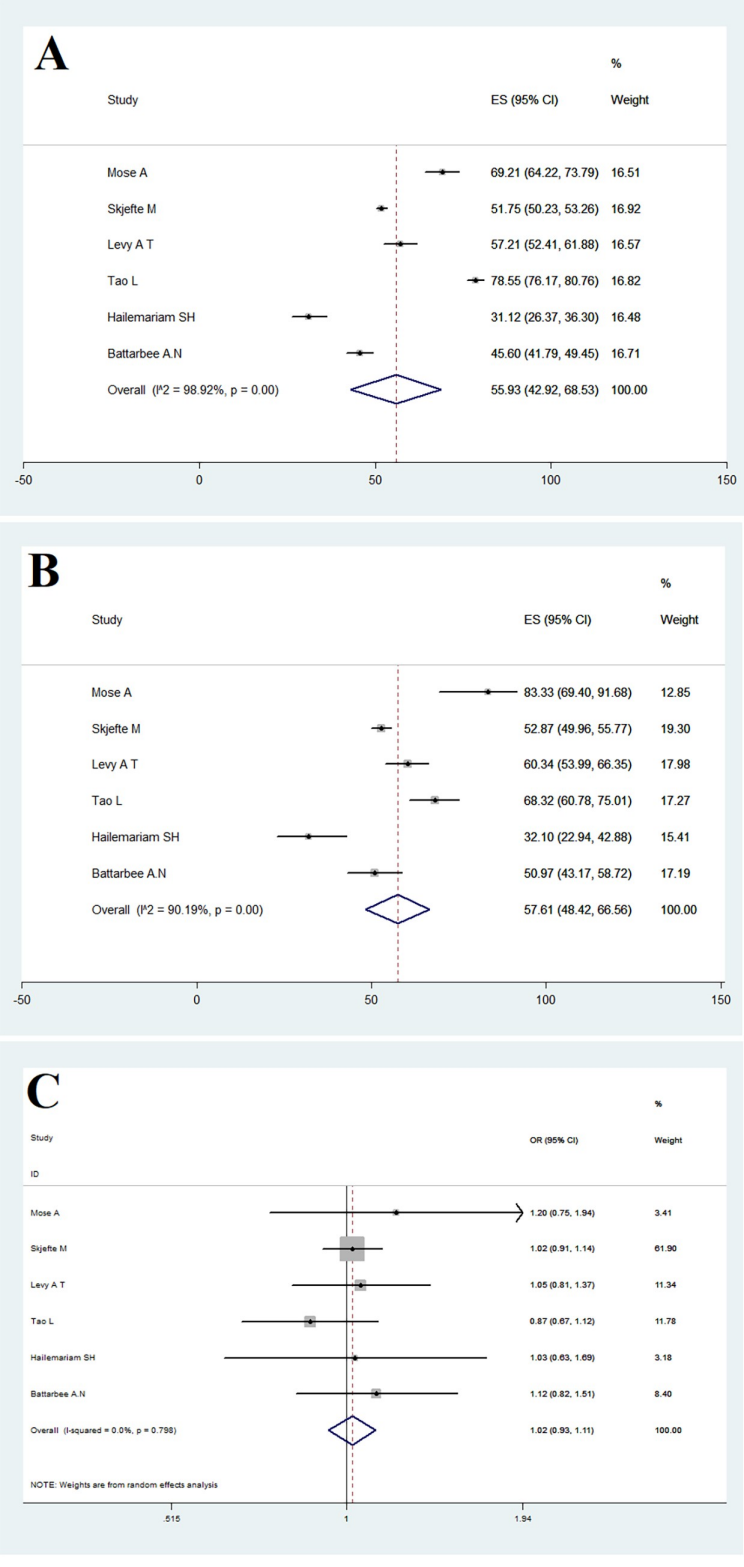
COVID-19 vaccine acceptance among fewer than 35 (A) and over 35 (B) pregnant women and the OR for COVID-19vaccine acceptance among fewer than 35 versus over 35 (C) pregnant women.

COVID-19 vaccine acceptance among pregnant women **fewer than** 35 and over 35 years was 55.93% (95% CI: 42.92–68.53) and 57.61% (95% CI: 48.42–66.56), respectively (Fig 4A and 4B). Furthermore, the relationship between COVID-19 vaccine acceptance for pregnant women and their age (**fewer than** 35 to over 35 years) was (OR = 1.02 [95% CI: 0.93–1.11]; Heterogeneity: I^2^ = 0, P = 0.798), which indicates that the relationship between vaccine acceptance in pregnant women and the age of pregnant women is not significant (Fig 4C).

### 3.6. The effect of gravid on COVID-19 vaccine acceptance

**Fig 5:**
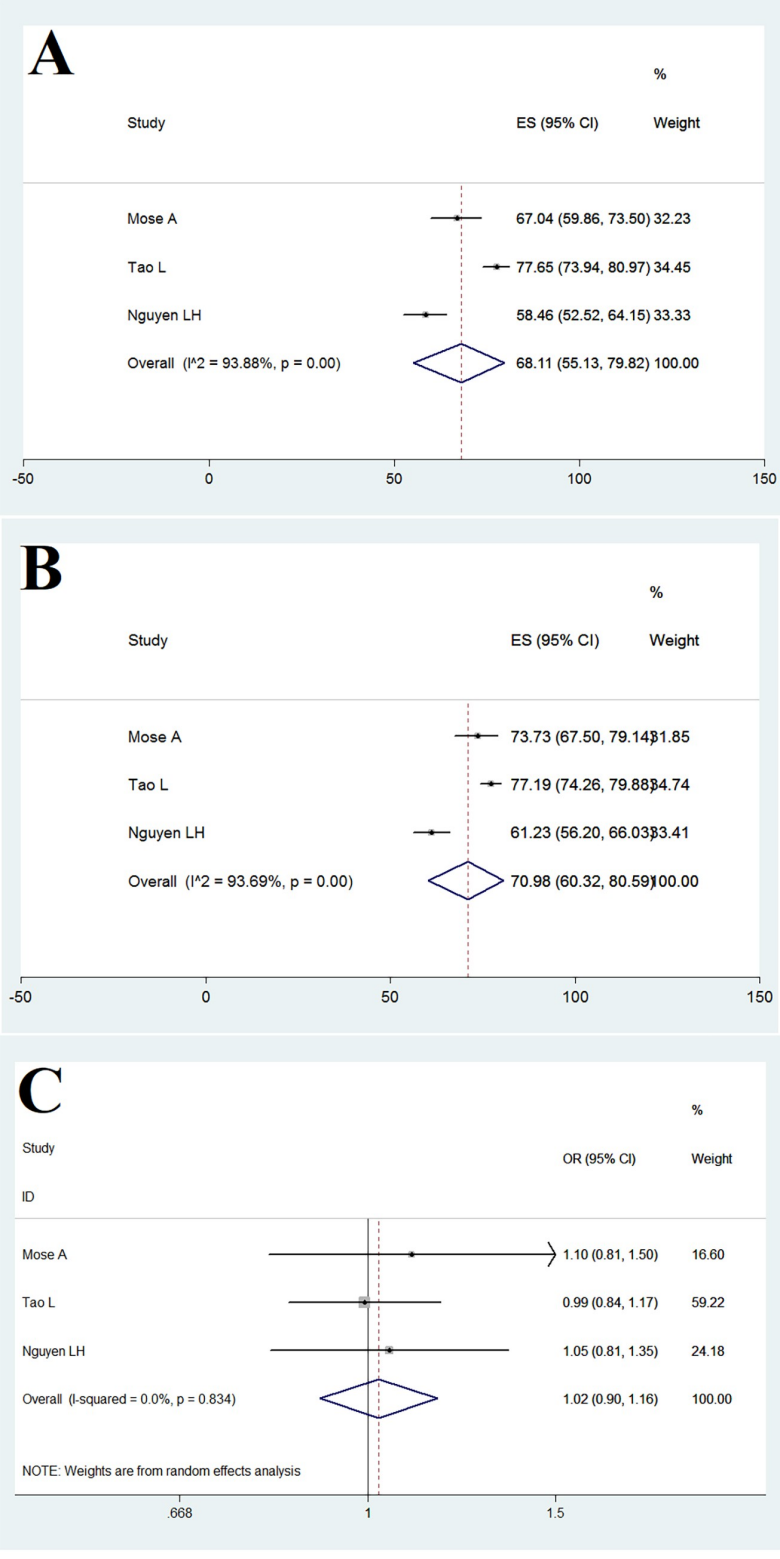
COVID-19 vaccine acceptance among primigraviad (A) and multigravida (B) pregnant women and the OR for COVID-19 vaccine acceptance primigraviad and multigravida pregnant women (C).

COVID-19 vaccine acceptance was 68.11% (95% CI: 55.13–79.82) among primigravida pregnant women and 70.98% (95% CI: 60.32–80.59) in multigravida women (Fig 5A and 5B). Moreover, the relationship between receiving COVID-19 vaccine in pregnant women and gravid was (OR = 1.02 [95% CI: 0.90–1.16]; Heterogeneity: I^2^ = 0, P = 0.834), which indicates that the relationship between vaccine acceptance in pregnant women and gravid in them is not significant (Fig 5C).

### 3.7. The effect of influenza vaccination history on COVID-19 vaccine acceptance

The prevalence of COVID-19 vaccine acceptance among pregnant women with a history of influenza vaccination was 61.13% (95% CI: 57.24–64.95) and without a history of influenza vaccination was 72.48% (95% CI: 70.18–74.72). In addition, the relationship between COVID-19 vaccine acceptance in pregnant women with a history of influenza vaccination and no history of influenza vaccination showed (OR = 0.87 [95% CI: 0.71–1.06]; Heterogeneity: I^2^ = 0, P = 0.574) (S1 Fig).

### 3.8. Meta-regression between vaccine acceptance variable and month of studies

Significant heterogeneity of vaccine acceptance for the month of the study was detected in meta-regression (P<0.001) (Table 2).

**Table 2:** Meta-regression analysis of the effect of the factors on vaccine acceptance in pregnant women.

Variable	Multivariable meta-regression		
% vaccine acceptance in pregnant women	Coefficient	P-value	[95% conf. Interval]
Publication month in 2021 years	53.15	<0.001	(44.48–61.82)

### 3.9. Publication bias

**Fig 6:**
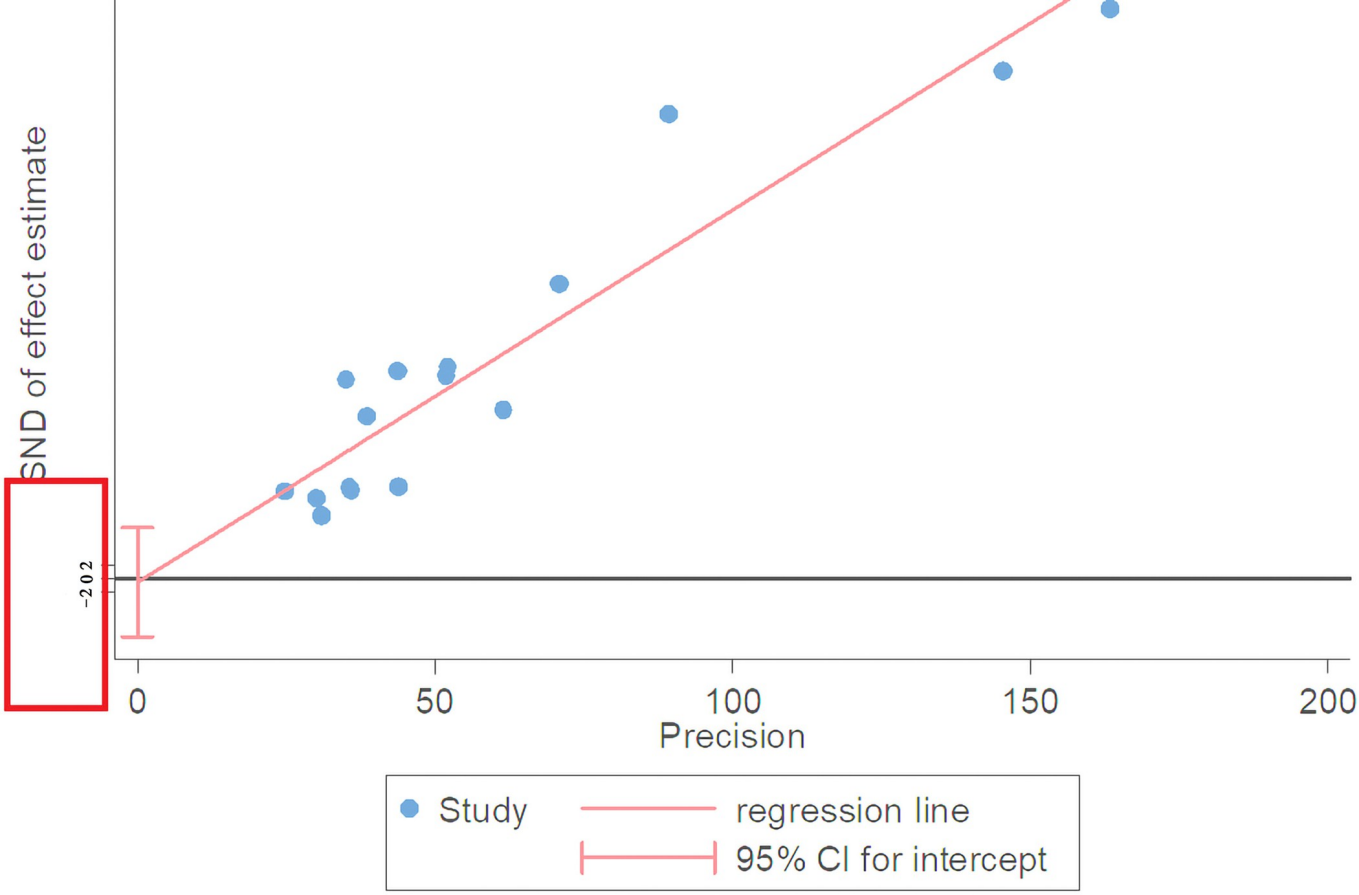
Egger diagram to investigate the bias in dissemination of results using the meta bias command for a meta-analysis of vaccine prevalence in pregnant women.

Egger’s test has more power to detect publication bias and is close to one according to the results of Kendall correlation coefficient in Begg’s test and is not statistically significant (p = 0.82), and is also insignificant in Egger’s test (p = 0.888) and its confidence interval ranges from -8.67 to 7.58, and since it includes zero, it indicates that no publication bias has occurred (Fig 6).

## 4. Discussion

This study is the first systematic review and meta-analysis on COVID-19 vaccine acceptance in pregnant women worldwide. The total prevalence of COVID-19 vaccine acceptance in pregnant women was estimated at 53.4%, with the lowest and highest rates being 37% and 84.5%, respectively. To investigate the cause of heterogeneity between studies, subgroup analysis was performed based on continent, data collection method and meta-regression based on the month of the study, and only meta-regression was significant based on the month of the study. The effect of some variables such as gravid, maternal age and history of influenza vaccination on COVID-19 vaccine acceptance was evaluated, which was not significant. It was not possible to examine other causes, but it seems that the possible explanation for these differences may be differences in access to health care services and awareness about the severity of COVID-19.

Widespread vaccination is the most promising strategy to end this global pandemic. COVID-19 vaccination started in February 2021 and the World Health Organization has approved more than three COVID-19 vaccines to reduce the incidence and potential threat of the disease [40–43]. Nevertheless, it is believed that the resistance of people, especially pregnant women, is high despite rapid preparation of the vaccine [44–47] and researchers attribute it to the extraordinary advances in the development of effective and safe vaccines against COVID-19 in a short time [48, 49]. In a systematic review and meta-analysis, COVID-19 vaccine acceptance in the general population was over 70%, and gender, education level, history of influenza vaccination, and trust in government were strong predictors of COVID-19 vaccination [50] but our estimate in the general population was much lower. We also compared COVID-19 vaccine acceptance among pregnant women with previous infectious diseases such as the H1N1 flu pandemic; COVID-19 vaccine acceptance in this study was higher than H1N1 vaccine acceptance in 2009 (47%) among pregnant women in the United States [51]. Other systematic reviews on the acceptance of influenza and pertussis vaccines during pregnancy shows that concerns about vaccine safety are one of the most important factors influencing the decision to receive the recommended vaccines during pregnancy [19, 52–55]. Thus, resistance to COVID-19 vaccine may be a limiting factor in global efforts to control the present pandemic, and have a negative impact on health and socio-economic aspects of society [55]. A previous systematic review also found that women were less likely to be vaccinated during the global influenza pandemic in 2009 [56]. This may be due to the fact that men show more risky behaviors than women [57]. Based on the Model of Health Belief, anticipated benefits (people who want to get the vaccine see a lot of anticipated benefits in receiving the COVID-19 vaccine to protect themselves and other people), cues to action (noteworthy predictors which elevated the intention to COVID-19 vaccine were suggestions provided by the Ministry of Health and GP or conducting the vaccination at workplace), and anticipated severity (severity of disease shows that the people that want to receive the vaccine see themselves as a person who is at high risk of notable pain or experiencing side effects if they are infected with COVID-19, as compared to people that do not want to receive the vaccine) were the most important predictors of the intention to get COVID-19 vaccine [58, 59]. Another reason for resistance to vaccine is the widespread anti-vaccination campaign in cyberspace. Johnson et al. argue that the internet has increased the audience of the anti-vaccine movement, and that it is possible that the explosive growth of anti-vaccination perspectives will hinder the development and acceptance of vaccines [60].

In the present study, the effect of some variables such as gravida, maternal age and history of influenza vaccine on COVID-19 vaccine was not significant. The results of a systematic review and meta-analysis demonstrated that high income, gender, marital status, influenza vaccine in the previous season, fear of COVID-19, confidence in the health system, higher education, chronic illness, and perceived risk are effective factors in COVID-19 vaccine acceptance [61]. The COVID-19 pandemic had great effects not only on vaccination against this sickness but also on readiness to get other vaccines, for instance against influenza. As a matter of fact, a huge anxiety for the upcoming winter is the combination of COVID-19 and influenza. Previous research has shown how an influenza pandemic can raise the acceptance of vaccination for seasonal influenza [62].

This study showed that sociodemographic variables have an effect on the rate of COVID-19 vaccine acceptance in Botswana. The older people (55 and older) had the highest acceptance rate for vaccine and this may be related to the fact that people in this age group pay attention to news provided by government sources, while younger groups often use social media and internet, which is full of unverified information and also the fact that this population has a higher risk of developing a severe case of the disease. On the contrary, a study in China demonstrated that middle-aged people (30–49 years) were more willing to receive the vaccine compared to other age groups. As authors mentioned, factors that influence willingness to receive the vaccine included paying much attention to the latest news related to the vaccine, among other factors [63].

Moreover, according to a systematic review and meta-analysis by Kilich et al., which examined factors influencing vaccination decisions among pregnant women, these women believe that vaccination can cause birth defects, injuries, long-term effects and anxiety [46], and this has important implications for public health messages about COVID-19 vaccination during pregnancy [64].

The present study showed that vaccine acceptance among pregnant women has increased significantly over time, which may be due to communication strategies such as positive orientations for action, being encouraged by close and trusted people such as doctors and religious leaders, sharing personal experiences, and peer pressure [65]. It should also be noted that similar to the influenza vaccine, the definitive recommendation of obstetricians to pregnant women to inject the vaccine is likely to increase the acceptance of COVID-19 vaccine [66, 67]. On the other hand, other evidences indicate that doctors’ advice for vaccination is the most important factor in the mother’s decision, regardless of geographical or social background. Furthermore, during the pandemic, pregnant women’s anxiety about the health of the fetus and its health has a negative impact on their well-being. Numerous studies have also shown that health professionals, including midwives, can reduce the level of anxiety in pregnant women by supporting them [8, 19, 68].

Changes in acceptance levels among pregnant women during a pandemic disease may be influenced by socio-demographic factors such as age, gender, and income status, individual factors such as personal beliefs, political views, risk perception, and social or organizational factors such as social media [69]; many factors affecting COVID-19 vaccine acceptance, such as geographical or socio-economic factors, hardly change and preliminary studies have not examined such variables.

Systematic strategies should be implemented to improve the acceptance of the COVID-19 vaccine among pregnant women. Previous research has shown that several combination interventions, including training sessions, easy access to vaccines, and vaccination rewards, can increase influenza vaccine acceptance [70]. We suggest that better health education and public health messaging can be used to address pregnant women’s concerns about fetal health and their own health. In addition, pregnant women should be informed about the benefits of protecting themselves, their family and friends after vaccination. We also recommend that national and individual interventions be performed to improve the COVID-19 vaccine acceptance among pregnant women in the future. At the national level, governments should instill public confidence in vaccines through scientific vaccine programs. In addition, governments need to be cautious and aware of potential anti-vaccine movements. In a part, it will be achieved by the integration of new emerging approaches and sciences to develop more reliable COVID-19 vaccine with minimum side effects in the future [71–74].

## 5. Limitations

One of the limitations of the present study is the lack of analysis in specific demographic subgroups due to the small number of articles.

## 6. Conclusion

The prevalence of COVID-19 vaccine acceptance in pregnant women was 53.46%, which was much lower than the general COVID-19 vaccination, but is consistent with the acceptance of other vaccines recommended in pregnancy such as influenza and tetanus, diphtheria and pertussis. Therefore, necessary interventions should be taken to increase the acceptance of the vaccine, address safety concerns and educate about it.

## Supporting information

S1 Checklist PRISMA 2009 checklist. (DOC)

S1 Fig COVID-19 vaccine acceptance among pregnant women with a history of influenza vaccination (A) without a history of influenza vaccination (B) and the OR for COVID-19 vaccine acceptance with a history of influenza vaccination and without a history of influenza vaccination pregnant women (C).
(PNG)
