# Supplementary figures and images for: COVID-19 vaccine acceptance among pregnant women worldwide: A systematic review and meta-analysis
Source: PLoS One. 2022 Sep 28;17(9):e0272273. doi: 10.1371/journal.pone.0272273 (PMC9518917; doi:10.1371/journal.pone.0272273)

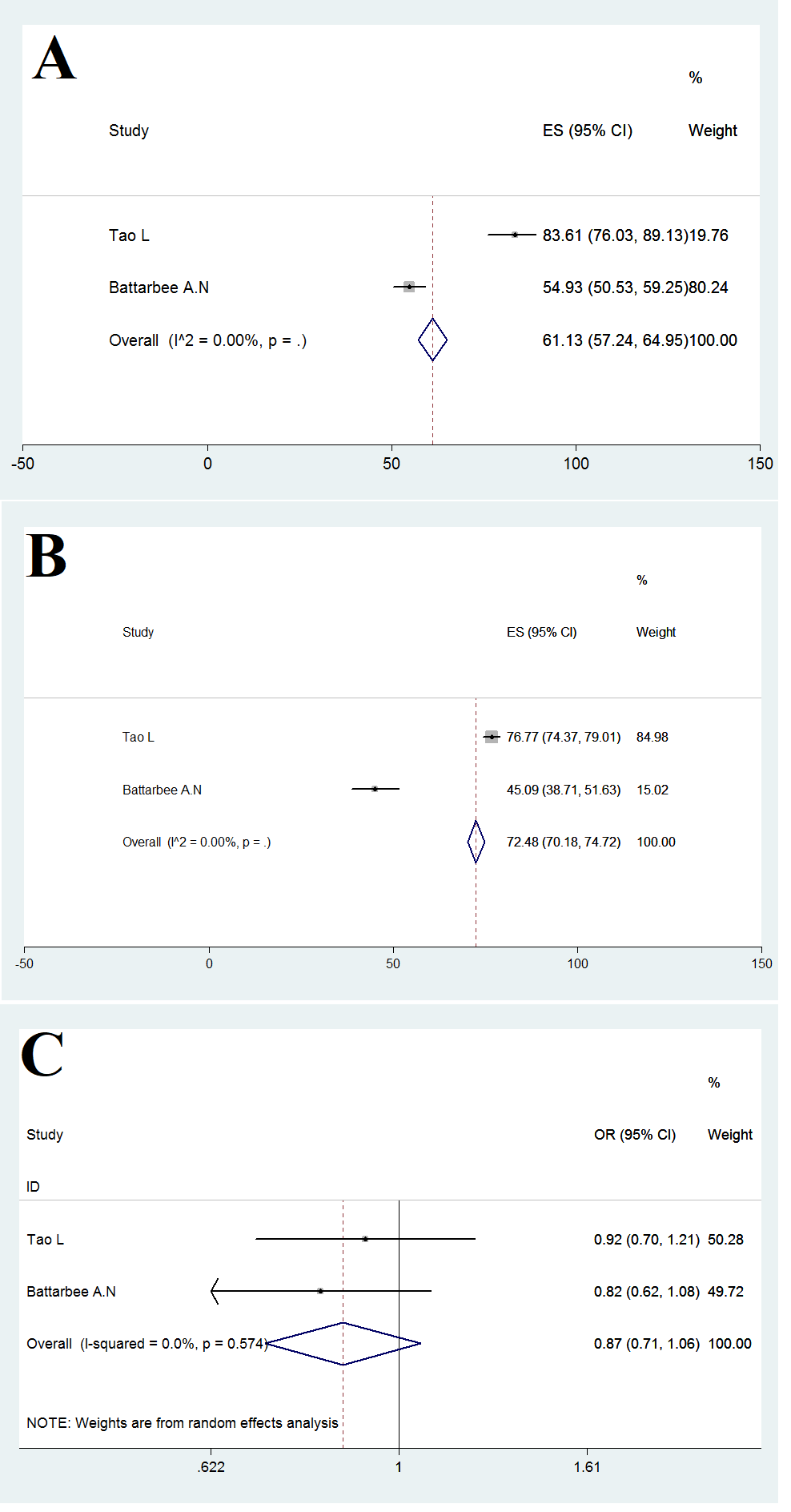

Supplement: S1 Fig — COVID-19 vaccine acceptance among pregnant women with a history of influenza vaccination (A) without a history of influenza vaccination (B) and the OR for COVID-19 vaccine acceptance with a history of influenza vaccination and without a history of influenza vaccination pregnant women (C). (PNG) [file pone.0272273.s002.png]
